# The Impact of a Mobile App on Participation in Cardiac Rehabilitation and Understanding Barriers to Success: Comparative Cohort Study

**DOI:** 10.2196/24174

**Published:** 2022-01-17

**Authors:** John T Rivers, Carla Smith, Ian Smith, James Cameron

**Affiliations:** 1 Queensland Cardiovascular Group St Andrew's Specialist Centre Brisbane Australia; 2 St Andrew's War Memorial Hospital Brisbane Australia; 3 St Vincent's Private Hospital Northside Brisbane Australia

**Keywords:** cardiac rehabilitation, digital health, smartphone app, Cardihab, participation rates, rehabilitation, cardiology, heart, app, barrier

## Abstract

**Background:**

Poor patient uptake of cardiac rehabilitation (CR) remains a challenge for multiple reasons including geographic, time, cultural, cost, and psychological constraints.

**Objective:**

We evaluated the impact on CR participation rates associated with the addition of the option of mobile app–based CR (Cardihab) for patients declining conventional CR.

**Methods:**

A total of 204 consecutive patients were offered CR following angioplasty; of these, 99 were in cohort 1 (offered conventional CR only) and 105 were in cohort 2 (app-based CR offered to those declining conventional CR). Patients in each cohort were followed throughout a 6-week CR program and participation rates were compared for both groups. Patients in cohort 2 declining both forms of CR were interviewed to assess reasons for nonparticipation.

**Results:**

CR participation improved from 21% (95% CI 14%-30%) to 63% (95% CI 53%-71%) with the addition of the app (*P*<.001). Approximately 25% (9/39) of the group declining the app-based program identified technology issues as the reason for nonparticipation. The remainder declined both CR programs or were ineligible due to frailty or comorbidities.

**Conclusions:**

Providing patients with the additional option of an app-based CR program substantially improved CR participation. Technology and psychological barriers can limit CR participation. Further innovation in CR delivery systems is required to improve uptake.

## Introduction

Although current guidelines recommend referral for cardiac rehabilitation (CR) following acute cardiac events, participation rates remain poor [1,2]. A recent estimate of the potential financial impact of increasing Australian CR participation rates from 30% to 50%-65% indicated net savings of Aus $46.7 million (US $33.9 million) to Aus $86.7 million (US $62.9 million) [2]. Clinical benefit is, however, more difficult to estimate, with some reviews questioning mortality benefit and others suggesting multicomponent CR programs may reduce overall mortality by up to 37% [3]. A Cochrane review of CR has confirmed lower rates of cardiovascular mortality and readmission among those who participate in exercise-based CR programs [4].

Conventional CR involves repeat attendance (usually 6-12 clinic visits) over a 6-week period. Previously described factors contributing to poor CR participation include issues of distance/transport, level of family support, gender roles, ethnicity, and cost [5-7]. Many currently available CR programs have not adapted to address these barriers. Additionally, changing patterns of treatment for acute events and much shorter hospital stays associated with more rapid return to work or home activities make prolonged conventional CR after an event less compatible with contemporary practice. Physical attendance may also be limited by a patient’s body image, gender, cultural beliefs, comorbidity, and psychological factors [5], and the requirement for social distancing during the COVID-19 pandemic.

To determine if app-based CR might help to overcome some of these barriers, we conducted an observational study on patients referred for CR in our facility. We hypothesized that offering the additional option of app-based CR for those patients declining conventional CR would increase participation rates compared to offering conventional CR alone. Information on reasons for nonparticipation in CR were collected to increase understanding of barriers and help identify ways to improve CR uptake.

## Methods

### Study Design and Participants

This study was conducted as a before (cohort 1) and after (cohort 2) design. During an initial 3-month recruitment period (cohort 1), consecutive patients undergoing angiography in two cardiac hospitals (St Andrew’s War Memorial Hospital and St Vincent’s Private Hospital, Brisbane, Australia) were monitored by an experienced cardiac nurse. Uptake and completion of a 6-week conventional, face-to-face CR program was documented for patients with acute coronary syndrome or elective intervention with percutaneous coronary intervention. Patients referred for cardiac surgery were excluded from the study due to the likely delayed uptake of CR.

Following completion of the conventional CR program by cohort 1, a second series of patients (cohort 2) was monitored throughout a subsequent 3-month recruitment period. Those patients in cohort 2 who declined conventional CR were offered the option of participating in a digital CR program delivered via smartphone app (Cardihab). Following completion of the 6-week CR program by cohort 2, CR participation rates were compared for both cohorts. Patients were evaluated based on the mode of CR in which they initially agreed to participate.

Review of the study design was undertaken by a representative of the UnitingCare Health Human Research Ethics Committee, who determined the study was an extension of an existing clinical service using a validated tool and full ethics committee review was not required. Informed consent was obtained from all patients.

### Description of App-Based CR Program

The app-based program consisted of an initial interview with a cardiac nurse, either face-to-face or remote, who admitted the patient to the web portal and collected baseline clinical data, including an assessment of prior physical activity levels and any constraints on physical activity. Patients with a compatible smart device (phone or tablet) were assisted to download either an iOS or Android version of the app. At first login, the 6-week program was activated and a series of daily and weekly tasks, based on the parameters entered during the admission interview, became visible in the app. Patients subsequently entered a variety of health measures, daily activity levels (type, intensity, and duration), and symptoms at regular intervals based on their specific clinical profile. The patient could visualize entered data in list or continuous graphical format. Activity reminders at scheduled intervals and encouragement messages were generated by the app.

Standardized education interventions were scheduled, with patient completion of these modules reported in the clinical portal. Patient understanding of the education modules was not assessed. Weekly telephone or video consultations were held between the patient and their cardiac nurse, who could review all app-derived data on relevant health measures, activity, and symptoms. Specific topics were scripted for weekly consultations along with discussion of clinical progress and barriers to completion of scheduled tasks.

### Barriers to Uptake of CR

Patients declining either form of CR participated in a semistructured qualitative interview with their cardiac nurse; the interview included a set of baseline questions, with the flexibility for the nurse to explore patient responses in greater detail as required. Patient-reported reasons for nonparticipation were recorded and categorized.

### Hospital Readmissions

For patients in cohort 2 (conventional CR, app-based CR, or no CR), the occurrence and cause of hospital readmissions within 12 months of the index cardiac event were retrospectively documented.

### Statistical Analysis

Rates of participation in CR in cohort 1 and cohort 2 were compared with a Chi-square test or Fisher exact test depending on numbers. Comparison of continuous variables employed the Mann-Whitney *U* test. In all comparisons, a *P* value of <.05 was considered statistically significant. Confidence intervals for proportions were calculated using the Wilson score interval. The study was not powered to evaluate changes in other clinical endpoints such as weight, waist circumference, or systolic blood pressure, and no statistical analysis of these endpoints was performed.

## Results

### Principal Findings

A total of 204 patients were offered CR following a percutaneous coronary intervention; this included 99 patients in cohort 1 (74% male; median age: males 70 years, females 73 years) and 105 patients in cohort 2 (75% male; median age: males 66 years, females 71 years; Table 1). There was no difference in the gender distribution between the two groups *(P=*.81), however, comparison of age distributions within each gender showed that males were significantly younger in cohort 2 (*P=*.005). There was no significant difference in female ages between the two groups (*P=*.16).

In cohort 1, a total of 21 patients (21%) undertook conventional CR, while in cohort 2, there were 43 patients (41%) that elected to undertake conventional CR (*P=*.002). Of the 62 patients (48 male) declining conventional CR in cohort 2, twenty-three (21 male) elected to participate in the app-based program. Overall, in cohort 2, there were 66 patients (63%) who undertook CR using either the conventional or app-based program. The increase in participation rate between cohort 1 and cohort 2 was statistically significant (*P*<.001).

As gender is a factor previously found to impact on CR participation [7], uptake by gender was evaluated. Participation by males in the CR program increased from 18% (n=13) in cohort 1 to 66% (n=52) in cohort 2 (*P<.*001). There was no significant difference apparent for females (8, 31% versus 14, 54%; *P=*.09). The increase in male participation arose from both an increased participation in the conventional program from 13 (18%) to 31 (39%), and a significant contribution from those taking up the app-based program (21/48, 44%).

**Table 1:** Summary of patient participation, age, and gender by mode of cardiac rehabilitation.

Variables	Cohort 1 (n=99)		Cohort 2 (n=105)		*P* value^a^
	Male	Female	Male	Female	
Patients approached, n (%)	73 (74)	26 (26)	79 (75)	26 (25)	.81
Median age (IQR)	70 (63-74)	73 (68-80)	66 (58-71)	71 (62-77)	Male: .005; female: .16
Conventional cardiac rehabilitation enrolled, n (%)^b^	13 (18)	8 (31)	31 (39)	12 (46)	.002
App-based cardiac rehabilitation enrolled, n (%)	N/A^c^	N/A	21	2	
Total cardiac rehabilitation uptake, n (%)^d^	13 (18)	8 (31)	52 (66)	14 (54)	*.*001

^a^*P* values for comparison between cohort 1 and cohort 2.

^b^Cohort one: 21 (21%, 95% CI 14%-30%); cohort two: 43 (41%, 95% CI 32%-51%). The Wilson score interval was used to calculate 95% CIs.

^c^N/A: not applicable.

^d^Cohort one: 21 (21%, 95% CI 14%-30%); cohort two: 66 (63%, 95% CI 53%-71%). The Wilson score interval was used to calculate 95% CIs.

Within cohort 2, patients participating in app-based CR were younger (median: 61 years versus 70 years, *P=.*005). Although the study was not powered to evaluate differences, trends were observed to higher weight (median: 90 kg versus 83 kg), higher BMI (median: 28.3 kg/m^2^ versus 26.5 kg/m^2^), and greater waist circumference (median: 105 cm versus 101 cm) in the app-based CR cohort.

There were 3 patients initially assigned to conventional CR who transitioned to app-based CR for completion of the program but they were counted as conventional CR based on their initial assignment. In addition, 2 patients in the conventional CR group and 1 in the app-based program commenced but did not complete CR.

### Barriers to Uptake of CR

Patients declining CR in cohort 2 (n=39) were interviewed to elicit reasons for nonparticipation (Table 2). Of note, 9 (23%) identified technology issues (either device or operator) as reasons for not taking up app-based CR. Psychosocial reasons for nonparticipation were also recorded for 9 (23%) patients. In addition, 11 patients did not commence CR due to further scheduled cardiac procedures, with most indicating they would consider CR following completion of interventions.

**Table 2:** Patient-reported reasons for declining participation in cardiac rehabilitation (n=39).

Reason	Number (%)
Further cardiac procedure scheduled	11 (26)
Psychosocial issues	9 (23)
Technical concerns (device or operator) regarding app-based cardiac rehabilitation	9 (23)
Comorbidities (Alzheimer, hearing difficulties)	3 (8)
Unable to be interviewed or living outside Australia	3 (8)
Completed cardiac rehabilitation previously and feel another program will not be useful	2 (5)

### Hospital Readmissions

Hospital readmissions within 12 months following the initial cardiac event for patients in cohort 2 are shown in Table 3. Readmissions were classified according to primary diagnosis as all-cause, cardiac-related, or bleeding-related. Although the study was not specifically designed to evaluate differences in readmission rates, cardiac readmission was observed to be very low (1/23, 4%) among the app-based (Cardihab) CR patients, considerably higher (13/43, 30%) for conventional CR patients, and 13% (5/39) for the patients who did not participate in any CR (*P=*.03). This may partly reflect a younger cohort in the app-based CR group (median age: 61 years, IQR 55-68 years versus conventional CR, median age: 70 years, IQR 62-74 years versus no CR, median age: 68 years, IQR 61-74 years).

**Table 3:** Hospital readmissions within 12 months of index cardiac event.

Readmission data		No cardiac rehabilitation	Conventional cardiac rehabilitation	App-based cardiac rehabilitation
**All participants**				
	Participants, n (% male)	39 (69)	43 (70)	23 (91)
	Age (years), mean (IQR)	68 (61-74)	70 (63-74)	61 (56-69)
**All readmissions**				
	Participants, n (% male)	10 (60)	21 (67)	5 (100)
	Age (years), mean (IQR)	65 (61-75)	69 (63-73)	68 (66-70)
	Proportion (95% CI)^a^	26 (15-41)	49 (35-63)	22 (10-42)
**Cardiac readmissions**				
	Participants, n (% male)	5 (60)	13 (77)	1 (100)
	Age (years), mean (IQR)^b^	66 (59-71)	69 (63-73)	68 (N/A^c^)
	Proportion (95% CI)^a^	13 (6-27)	30 (19-45)	4 (1-21)
**Bleeding-related readmissions**				
	Participants, n (% male)	3 (67)	2 (100)	0 (0)
	Age (years), mean (IQR)^b^	77 (N/A)	77 (N/A)	N/A
	Proportion (95% CI)^a^	8 (3-20)	5 (1-15)	0 (0-14)

^a^Confidence intervals (95%) shown for proportions were calculated using the Wilson score interval.

^b^No IQR is provided where the number of cases is less than 5.

^c^N/A: not applicable.

## Discussion

### Principal Findings

Providing the additional option of an app-based CR program was associated with an increase in overall CR participation rate of 42%, from 21% (21/99) in cohort 1 to 63% (66/105) in cohort 2. The improved uptake in CR following the addition of an option for app-based CR suggests that a significant proportion of patients will benefit from the convenience and flexibility of a remotely delivered program.

A remote digital CR program using a smartphone app that communicates with a clinician portal can automate aspects of care delivery and standardize much of the content of conventional CR while tailoring a specific program for individual patient needs. A previous randomized controlled trial confirmed that an app-based program can deliver CR with at least comparable efficacy to conventional CR [8]. Other trials of digital CR programs using a mobile app have demonstrated improved participation and adherence to CR, improved exercise capacity [9], and reduced readmission rates over 12 months [10]. As patients may prefer conventional, digital, or blended models of care for a complex array of reasons, it is important to consider patient treatment preferences to help optimize completion rates. A recent Australian position statement addressing secondary prevention during the COVID-19 pandemic strongly recommended the use of eHealth strategies to continue delivering evidence-based therapies to patients [11].

Recent reviews of the potential of smart device apps in the long-term management of chronic diseases have concluded that apps have substantial potential to improve health outcomes [12-15]. One review noted, however, that significant improvements were recorded in 50% of the interventions that were solely app-based compared with 100% of the interventions where the app was a component of a clinical team management protocol [13]. The emphasis on continued close involvement by the clinical CR team is a likely key success factor for app-based CR and the inclusion of digital health apps should be considered as another tool in program delivery, rather than disruptive, for this model of care. Qualitative feedback from the app-based group in this study suggested patients placed a high value on continued monitoring from their clinical team.

Many patients attending CR sessions identify group dynamics and social interaction as positive motivating factors and will continue to select conventional, face-to-face CR as a preferred option. Future integration of virtual, private social media groups into app-based CR may reduce this preference effect. A recent randomized study using the WeChat social media platform to deliver CR demonstrated improved exercise capacity at 2 and 6 months, improvements at 12 months in coronary artery disease knowledge score, lower systolic blood pressure and heart rate, lower total and LDL cholesterol, and higher medication adherence in the digital CR group [16].

### Addressing Barriers to CR Uptake

The provision of an app-based CR program can help overcome a number of the barriers associated with conventional CR, particularly the need for patients to travel long distances to attend, with the associated costs of transport and parking, as well as the barrier posed by social distancing restrictions implemented as a consequence of the COVID-19 pandemic. It also helps alleviate the time constraints associated with attending face-to-face CR, and has the capability to address cultural barriers associated with language and gender roles by providing programs in multiple languages, and the option for care coaches and family members to join the device program.

As previous experience [8] indicated that technology issues might be a significant factor limiting uptake of app-based CR, patients were interviewed to understand reasons for nonparticipation in CR. Technology issues were a substantial barrier for approximately 25% (9/39) of the cohort unable to undertake the app-based program, due to device issues or operator problems. Device issues included phones without internet connection or capability, problems with operating platforms, and older devices that did not support recent software versions. Operator problems included reluctance to use any type of app solution, difficulties with app downloads and account details, and a few instances of difficulty with manual data entry. Ongoing technology coaching by the CR team was required in some cases, supported by online video tutorials. Therefore, device suitability and a patient’s technical literacy are important considerations for patient selection. Provision of loan or rental devices could help overcome device suitability issues. A worthwhile alternative to clinical staff providing technical support, suggested by patients to facilitate the adoption of a digital pulmonary rehabilitation program, is the creation of a peer-to-peer social learning environment to support patients with technology and motivation [17]. This approach could be considered for future app-based programs.

Anxiety or depression is present in at least 15% to 20% of patients after an acute cardiovascular event and this may be a barrier to the behavior change and adoption of a healthier lifestyle represented by CR [18]. These factors may also predispose patients to failure to complete CR, with a compounding effect on longer-term adverse outcomes [19]. It is likely that similar psychosocial factors contributed to the approximately 25% (9/39) of the cohort who declined taking up either modality of CR. App-based CR may alleviate some of the anxiety associated with conventional CR as activity levels can be customized and completed in private. Depression screening tools may also be incorporated into digital CR programs.

### Hospital Readmissions

Readmission after major cardiac events is a significant and costly problem [1,3], with 30-day rates estimated between 6%-27% and 12-month rates estimated at 20%-30% [1,20]. This study was not powered to address differences in readmission rates but the very low rate of 4% observed for app-based CR compared to other groups is hypothesis generating. The younger and predominantly male app-based CR cohort may have had fewer comorbidities, which could partly explain this observation. This raises the important possibility of risk stratifying interventions to target higher risk groups and specifically measuring readmission outcomes in app-based CR compared to conventional approaches. Accurate assessment of risk status using a validated tool such as the PEGASUS-TIMI 54 score [21] in a larger prospectively designed trial employing app-based CR delivery should be considered.

### Limitations

The findings of this work must be viewed in light of the study’s limitations. As the effectiveness of the digital CR program had been tested in a previous randomized controlled trial [8], this study was intended to assess the real-world efficacy of a blended model of delivering CR. As a before-and-after study design without a control group, the outcomes are subject to biases associated with variations in patient characteristics and circumstances between cohort 1 and cohort 2. Although the basic distribution of males and females is similar in both cohorts, analysis suggests that the age of males in cohort 2 is significantly lower than that of males in cohort 1. This age disparity may account for some of the outcome differences noted in these two groups, particularly in terms of comorbidities that may have impacted hospital readmissions. Furthermore, the relatively small population involved in this study places significant limitations on any analysis involving subgroups.

### Conclusion

A clinically validated app-based CR program can improve CR participation and should be considered as a standard component of a CR service, particularly for those patients who find conventional CR impractical, inconvenient, or unappealing. Study summary slides are available in Multimedia Appendix 1. Further trials are needed to assess the value of app-based risk factor modification on long-term clinical outcomes across the spectrum of coronary artery disease, from early diagnosis to long-term secondary prevention.

## Appendix

Multimedia Appendix 1 Study summary.

## Abbreviations

CR: cardiac rehabilitation
