# Supplementary material for: The Impact of a Mobile App on Participation in Cardiac Rehabilitation and Understanding Barriers to Success: Comparative Cohort Study
Source: JMIR Cardio. 2022 Jan 17;6(1):e24174. doi: 10.2196/24174 (PMC8804955; doi:10.2196/24174)
Supplement: Multimedia Appendix 1 [file cardio_v6i1e24174_app1.pdf]

The Impact of a Mobile App on Participation in Cardiac Rehabilitation and Understanding Barriers to Success: Comparative Cohort Study

- John T Rivers [1,2,3,] MBBS,FCSANZ; Carla Smith [1], RN; Ian Smith[2], PhD; James Cameron MBBS,FCSANZ [1,2]
    - 1 Queensland Cardiovascular Group, St Andrew’s Specialist Centre, Brisbane, Australia
    - 2 St Andrew’s War Memorial Hospital, Brisbane, Australia
    - 3 St Vincent's Private Hospital Northside, Brisbane, Australia Corresponding

Queensland Cardiovascular Group

Cardihab Pty Ltd, Level 3, 315 Brunswick Street, Fortitude Valley, QLD 4006.
e: info@cardihab.com | w: cardihab.com| ABN: 20 610 971 910|

Background and Objectives

Queensland Cardiovascular Group

- Background: Poor patient uptake of cardiac rehabilitation (CR) remains a challenge for multiple reasons including geographic, time, cultural, cost and psychological constraints.

- Objective: We evaluated the impact on CR participation rates associated with the addition of the option of smartphone enabled app-based CR (SmartCR) for patients declining conventional CR.

Methods

Queensland Cardiovascular Group

- 204 consecutive patients were offered CR post angioplasty, 99 in cohort 1 (offered conventional CR only) and 105 in cohort 2 (app-based CR SmartCR offered to those declining conventional CR).
- Patients in each cohort were followed throughout a 6-week CR program and participation rates were compared for both groups.
- Patients in cohort 2 declining either form of CR were interviewed to assess reasons for non-participation.

SmartCR Patient app

Queensland Cardiovascular Group

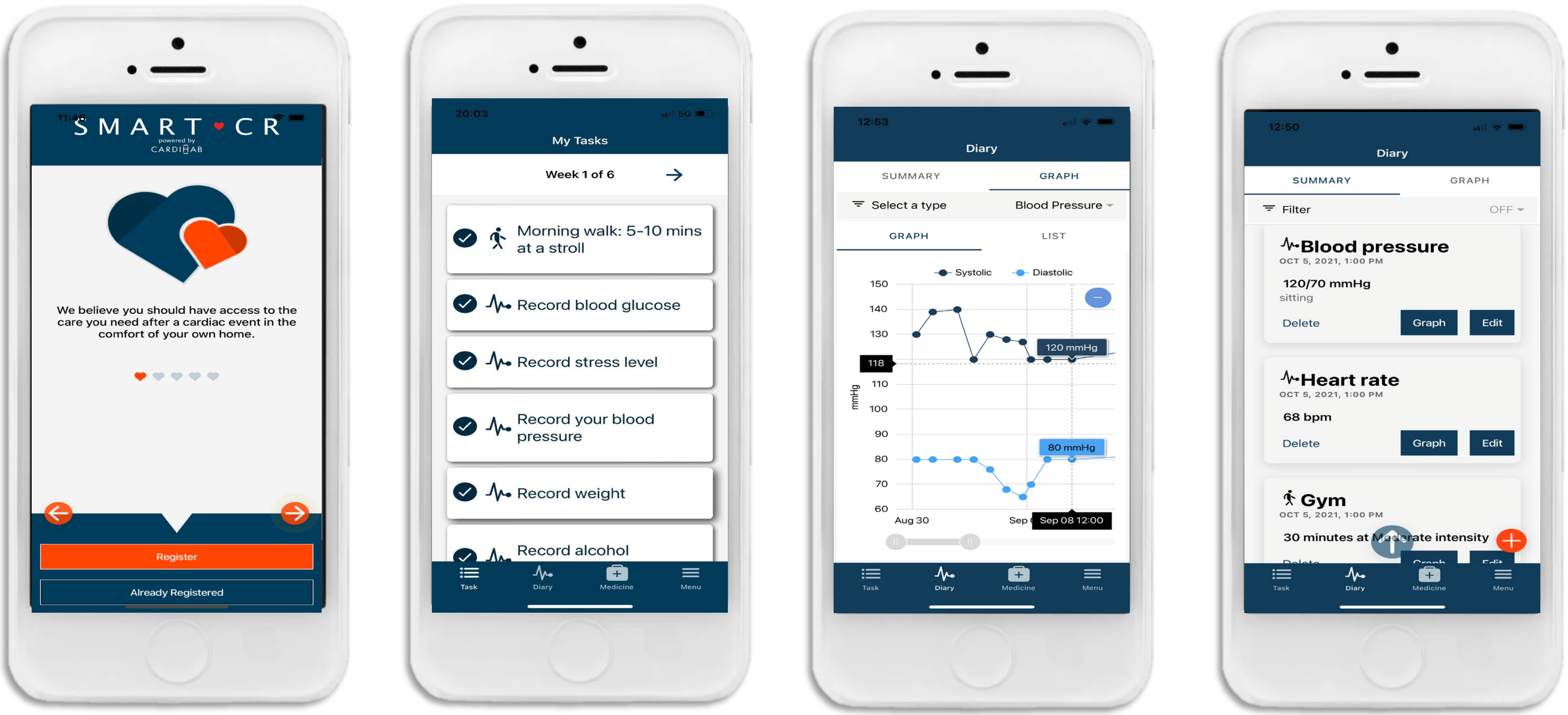

CARDIHAB

Cardihab Pty Ltd, Level 3, 315 Brunswick Street, Fortitude Valley, QLD 4006.
e: info@cardihab.com | w: cardihab.com| ABN: 20 610 971 910|

Patient Participation

Queensland Cardiovascular Group

CR participation improved from

Cohort 1: 21% (95% CI 14%-30%)

to

Cohort 2: 63% (95% CI 53%-71%)

with the addition of the

smartphone enabled app (P<.001).

Summary of patient participation by mode of cardiac rehabilitation

	Cohort 1 (n=99)		Cohort 2 (n=105)		
	Male	Female	Male	Female	
Total CR uptake	21 (21%, CI:14%-30%)		66 (63%, CI: 53%-71%)		p<0.001*
	13 (18%)	8 (31%)	52 (66%)	14 (54%)	

*p-values for comparison between phase 1 and phase 2. CI, 95% Confidence intervals calculated using the Wilson score interval. IQR, interquartile range; CR, cardiac rehabilitation

Overall CR Participation Rates

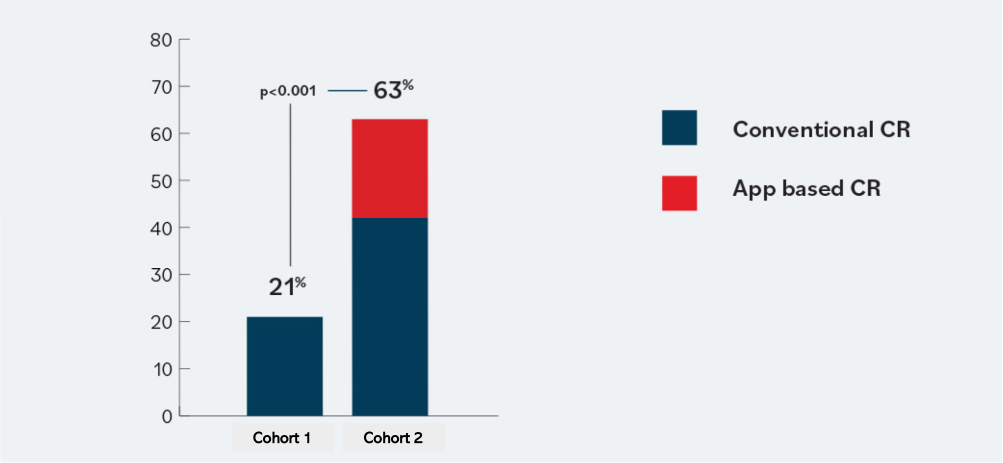

CARDIHAB

Cardihab Pty Ltd, Level 3, 315 Brunswick Street, Fortitude Valley, QLD 4006.
e: info@cardihab.com | w: cardihab.com| ABN: 20 610 971 910|

Patient reported reasons for declined participation

Approximately 25% of the group declining the app-based program identified technology issues as the reason for non-participation.

The remainder declined both CR programs or were ineligible due to frailty or co-morbidities.

Patient-reported reasons for declining participation in CR (n = 39)

Reason	Number (%)
Further cardiac procedure scheduled	11 (26%)
Psychosocial issues	9 (23%)
Technical concerns (device or operator) re app-based CR	9 (23%)
Comorbidities (Alzheimer's; hearing difficulties)	3 (0.08%)
Unable to be interviewed or living outside Australia	3 (0.08%)
Completed CR previously and feel another program won't be useful	2 (0.05%)

Hospital Readmissions

Queensland Cardiovascular Group

Readmission after major cardiac events is a significant and costly problem [1, 3] with 30-day rates estimated between 6-27% and 12-month rates estimated at 20-30% [1, 20].

The current study was not powered to address differences in readmission rates but the very low rate of 4% observed for app-based CR compared to other groups is hypothesis generating.

Table 3: Hospital readmissions within 12 months of index cardiac event

		No CR	Conventional CR	App-based CR
**Patients (Phase 2)**	n	39 (M: 69%)	43 (M: 70%)	23 (M: 91%)
	Age (IQR)	68 (61-74)	70 (63-74)	61 (56-69)
**All readmissions**	n	10 (M: 60%)	21 (M: 67%)	5 (M: 100%)
	Age (IQR)	65 (61-75)	69 (63-73)	68 (66-70)
	Proportion (%)	26% (15%-41%)	49% (35%-63%)	22% (10%-42%)
**Cardiac readmissions**	n	5 (M: 60%)	13 (M: 77%)	1 (M: 100%)
	Age (IQR)	66 (59-71)	69 (63-73)	68 (n/a)
	Proportion (%)	13% (6%-27%)	30% (19%-45%)	4% (1%-21%)

IQR, interquartile range; F2F, face to face. No IQR is provided where the number of cases is less than 5. Confidence intervals (95%) shown for proportions were calculated using the Wilson score interval.

CARDIHAB

Conclusions

- Providing patients with the additional option of an app-based CR program substantially improved CR participation.
- Technology and psychological barriers can limit CR participation.
- Further innovation in CR delivery systems is required to improve uptake.
- (JMIR Cardio 2021;0(0):e0) doi: 10.2196/24174)
